# Tert-Butyl Hydroperoxide in Human Adult Mesenchymal Stem Cells Isolated from Dermis: A Stress-Induced Premature Senescence Model

**DOI:** 10.3390/cells14191563

**Published:** 2025-10-08

**Authors:** Luca Pampanella, Giovannamaria Petrocelli, Provvidenza Maria Abruzzo, Riccardo Tassinari, Beatrice Bassoli, Rossella Sgarzani, Margherita Maioli, Carlo Ventura, Silvia Canaider, Federica Facchin

**Affiliations:** 1 Department of Medical and Surgical Sciences (DIMEC), University of Bologna, Via Massarenti 9, 40138 Bologna, Italy; luca.pampanella2@unibo.it (L.P.); giovannam.petrocell2@unibo.it (G.P.); provvidenza.abruzzo2@unibo.it (P.M.A.); beatrice.bassoli2@studio.unibo.it (B.B.); rossella.sgarzani2@unibo.it (R.S.); carlo.ventura@unibo.it (C.V.); federica.facchin2@unibo.it (F.F.); 2 National Laboratory of Molecular Biology and Stem Cell Bioengineering of the National Institute of Biostructures and Biosystems (NIBB) c/o Eldor Lab, Via Corticella 183, 40129 Bologna, Italy; riccardo.tassinari.rt@gmail.com; 3 Department of Biomedical Sciences, University of Sassari, Viale San Pietro 43/B, 07100 Sassari, Italy; mmaioli@uniss.it; 4 IRCCS Azienda Ospedaliero-Universitaria di Bologna, Via Massarenti 9, 40138 Bologna, Italy

**Keywords:** dermal mesenchymal stem cells, tert-butyl hydroperoxide, hydrogen peroxide, stress-induced premature senescence (SIPS)

## Abstract

Stem cell (SC)-based therapy exploits the ability of cells to migrate to damaged tissues and repair them. In this context, there is a strong interest in the use of mesenchymal stem cells (MSCs), multipotent SCs that are easy to obtain and are able to differentiate into various cell lineages. However, MSCs undergo cellular senescence during in vitro expansion, and may also become senescent in vivo, influenced by multiple molecular, cellular, and environmental interactions. Therefore, the development of in vitro cell models is crucial to study the mechanisms underlying senescence in MSCs. This study aimed to investigate the effects of tert-butyl hydroperoxide (t-BHP) as a senescence inducer in human dermal MSCs (hDMSCs), a promising tool for tissue repair. t-BHP induced a pro-senescent effect on hDMSCs greater than hydrogen peroxide (H_2_O_2_), as evidenced by ROS production, DNA damage, cell cycle arrest, inhibition of cell proliferation, changes in cellular and nuclear morphology, and cytoskeletal reorganization, as well as the increase in other senescence markers, including senescence-associated β-galactosidase (SA-β-Gal)-positive cells, and senescence-associated secretory phenotype (SASP). These results indicate that t-BHP could be a promising compound for inducing stress-induced premature senescence (SIPS) in hDMSCs, providing a valuable tool to investigate this process and evaluate the efficacy of senolytic compounds.

## 1. Introduction

Stem cell (SC) therapy is a regenerative medicine approach that takes advantage of the different SC characteristics, such as self-renewal and differentiation, to treat various human diseases, including neurological disorders, cardiovascular conditions, pulmonary dysfunctions, endocrine diseases, and skin burns [1]. Human mesenchymal stem cells (hMSCs) have been widely used in this context, also because they can be isolated in large quantities from specific body sources and their use is associated with only a few ethical concerns [1,2].

In 2005, MSCs were first identified in the human dermis and subsequently in the same tissue of other species, including mice and geese [3,4]. They grew in adhesion to the plastic support and showed typical MSC properties, so they were called “dermal mesenchymal stem cells” (DMSCs) [3]. DMSCs appeared to be involved in embryonic and epidermal development, as well as in the hair follicle cycle [3,4]. They also showed significant therapeutic potential in wound healing, promoting skin wound closure and angiogenic process in mice, and reducing inflammation by converting pro-inflammatory type 1 macrophages (M1) into pro-reparative M2 macrophages [5,6]. Moreover, DMSCs stimulated liver activity and regeneration, improving liver fibrosis and survival in mice exposed to radiation [7]. Finally, DMSCs could differentiate into insulin-producing cells, making them a potential tool for the treatment of type I diabetes [8]. The therapeutic role of DMSCs is mainly based on their immunomodulatory potential, characterized by a reduced incidence and severity of acute graft-versus-host disease (GVHD) after cell transplantation, linked to a reduced proliferation of helper T lymphocytes produced by the spleen and increased activity of regulatory T lymphocytes [9,10].

hDMSCs, like other SCs, undergo senescence during in vitro expansion. Chronic senescence is a cellular state characterized by an irreversible cell cycle block, which not only affects the ability of MSCs to divide but also impairs their differentiation potential and survival [11,12,13]. In addition, senescence reduces the MSC ability to migrate towards damaged tissues and promotes the production of “senescence-associated secretory phenotype” (SASP) factors, able to induce an inflammatory state in MSC-transplanted patients [13]. Together, all these senescence-associated characteristics could compromise MSCs’ therapeutic potential. Moreover, the transplantation context influences SCs’ senescence and consequently their therapeutic efficacy. For example, diseases such as obesity, diabetes, metabolic syndromes, chronic inflammatory states, and the pathologies related to aging itself can promote stress conditions and inflammation, thus accelerating the senescence of transplanted cells [11,13].

Therefore, in order to improve the therapeutic outcome of SC transplantation, it becomes essential to delve into the causes and mechanisms of senescence, as well as to identify possible strategies to counteract or slow down this process. To study cellular senescence in vitro, it is useful to use cells cultured for many passages (replicative senescence) or cells induced to enter in a state of senescence (stress-induced premature senescence (SIPS)), thus obtaining cell models to better understand the process.

SIPS can be induced by using different physical or chemical stimuli such as UV, γ radiation, hydrogen peroxide (H_2_O_2_), ethanol, D-galactose, doxorubicin, and tert-butyl hydroperoxide (t-BHP) [14,15].

H_2_O_2_, which is easily available and very cheap, has been widely used to promote and study the senescence in dermal fibroblasts [16,17,18], and in numerous types of MSCs, including hDMSCs, where it has been shown to induce senescence in a dose- and time-dependent manner [15,19]. In dermal fibroblasts, H_2_O_2_ was often used in the range of 150–400 μM for 1–2 h (h) to induce a senescent phenotype [20,21]. Also, in DMSCs, the effects of a wide range of concentrations, from 0.1 to 500 μM, were studied [22]. With the increase in H_2_O_2_ concentration, these cells appeared particularly predisposed to apoptosis, a process that may represent the primary mechanism of defense of the hDMSCs against stress [23].

t-BHP is another molecule used to study oxidative stress and senescence in vitro in different cell types [24,25,26,27]. It has been demonstrated that t-BHP depletes the cellular pool of glutathione (GSH), an antioxidant molecule, leading to an increase in reactive oxygen species (ROS) production, which in turn causes damage to cell membrane and macromolecules such as DNA, proteins, and lipids [24,25]. Moreover, in human fibroblasts, t-BHP has shown the ability to induce a senescent phenotype; it increased the expression of the cyclin-dependent kinase inhibitor 1A (CDKN1A, alias p21) and tumor protein P53 (p53) with consequent blocking of the proliferation process, stimulated the production of senescence-associated β-galactosidase (SA-β-Gal), and promoted the release of SASP components such as matrix metalloproteinase (MMP) 1, MMP3, serpin family B member 2 (SERPINB2), interleukin (IL)-1A, epidermal growth factor (EGF), and IL-8 [24]. A similar effect has also been observed in endothelial cells, highlighting a dose-dependent effect of t-BHP. At high concentrations, this molecule triggered apoptosis or even necrosis, while, at intermediate doses, it promoted the onset of senescence, as evidenced by SA-β-Gal production and reduced telomerase activity [25]. Moreover, a pro-senescent effect of t-BHP has been demonstrated in certain types of SCs, as neuronal SCs (NSCs); in these cells, t-BHP reduced the expression of proliferation marker protein Ki-67, leading to an increase in the number of cells in the G1 phase, and induced the production of the SA-β-Gal enzyme, high levels of p16^INK4a^ (also named cyclin-dependent kinase inhibitor 2A—CDKN2A), and low levels of cyclin D1 (CCND1) and retinoblastoma protein (pRb) [28]. Similar results have been reported in studies on nucleus pulposus-derived MSCs (NPMSCs), where t-BHP induced a high production of ROS and SASP components such as IL-1B, IL-6, and IL-8, as well as the activation of the Jun N-terminal kinase (JNK)/c-Jun pathway [29]. In the literature, a wide range of concentrations and treatment times were reported for this molecule, which would explain the different effects observed in cells; these effects also depend on the sensitivity of the different cellular models to t-BHP treatment [15,24,25,26,27,28,29,30,31].

Given the therapeutic role of hDMSCs and the importance of identifying in vitro models to study the senescence process of MSCs, the aim of this research was to investigate the pro-senescent effects of different concentrations of t-BHP in hDMSCs, assessed 48 h after the end of a 2 h treatment and compare them with those of H_2_O_2._ To this purpose, viability, ROS production, proliferation, cellular and nuclear morphology, cytoskeletal organization, and senescence-associated markers were analyzed in hDMSCs stressed with both compounds.

## 2. Materials and Methods

### 2.1. Harvesting and Cultures of hDMSCs

Human DMSCs were obtained from the dermis of 3 healthy donors after informed consent. The isolation protocol was adapted from the experimental procedure described by Ferroni and co-workers [32]. Briefly, the dermis was first separated from the other skin tissues and carefully washed to remove blood clots and cell debris. Then, small biopsies of 3–5 mm diameter were harvested, minced by scalpel, and digested with a solution of Collagenase I (Cat. N. C0130; Sigma-Aldrich Co., St. Louis, MO, USA) and II (Cat. N. C6885; Sigma-Aldrich Co., St. Louis, MO, USA) (ratio 1:1 *v*/*v*) for 2 h and 30 min (min). Afterwards, biopsies were plated in 25 flasks (Corning Incorporated, Corning, NY, USA) and cultured in Dulbecco’s modified Eagle’s medium—1 g/L of glucose (L-DMEM, Cat. N. ECM0749L; Euroclone, Pero, MI, Italy) supplemented with 10% fetal bovine serum (FBS, Cat. N. 10270106; Gibco, Waltham, MA, USA), L-glutamine, and antibiotics (penicillin/streptomycin solution, both 1%, Cat. N. ECB3001D; Euroclone, Pero, MI, Italy—and were maintained in standard culture conditions at 37 °C with 5% carbon dioxide (CO_2_) in a humidified atmosphere. The culture medium was replaced after seven days. After an additional week, cells were detached by using a trypsin-EDTA solution (Cat. N. BE02-007E; Lonza, Basel, Switzerland) and were used for expansion and/or experimental investigations. For each analysis, hDMSCs were seeded at a defined density in suitable plastic support (Corning Incorporated, Corning, NY, USA) and incubated in standard condition for 24 h before treatment. Experiments were carried out using hDMSCs at the 3rd–5th passage of culture and were repeated in biological triplicate (*n* = 3).

This study was conducted according to the Declaration of Helsinki and was approved by the Ethical Committee of Romagna (C.E.ROM., Sede Operativa c/o IRCSS Istituto Romagnolo per lo Studio dei Tumori “Dino Amadori”—IRST S.r.l.—Via Piero Maroncelli 40, 47014 Meldola (FC), Reg. emendamenti n° 5789, protocol n° 4099/2024, I.5/14, 18 July 2024).

### 2.2. hDMSC Characterization: Surface Markers’ Expression and Differentiation Potential

To characterize the phenotype of hDMSCs, the expression of surface markers was evaluated. Briefly, cells were detached from 75 cm^2^ flasks (Corning Incorporated, Corning, NY, USA), washed in phosphate buffered saline (PBS, Cat. N. ECB4004L; Euroclone, Pero, MI, Italy), and fixed with 4% formaldehyde (Cat. N. 1.00496; Sigma-Aldrich Co., St. Louis, MO, USA) for 10 min at room temperature (RT). Then, cells were incubated for 30 min at 4 °C in darkness with the following antibodies: CD90-fluorescein isothiocyanate (FITC) (Cat. N. 11-0909-42), CD44-FITC (Cat. N. 11-0441-82), CD105-phycoerythrin (PE) (Cat. N. 12-1057-42), CD73-PE (Cat. N. 12-0739-42), CD45-peridinin chlorophyll (PerCP) (Cat. N. 46-0459-42), and CD34-PerCP (Cat. N. 46-0349-42). All monoclonal antibodies were purchased from eBioscienceTM (Thermo Fisher Scientific, San Diego, CA, USA). After one wash with PBS, a total of 10,000 events (viable cells) were acquired using the CytoFLEX S Flow cytometer (Beckman-Coulter Inc., Brea, CA, USA). Data analysis was performed using FlowJo v10.8 software (Tree Star, Ashland, OR, USA).

The adipogenic potential of hDMSCs was assessed by seeding them at a density of 9000 cells/cm^2^ in 48-well plates (Corning Incorporated, Corning, NY, USA). Adipogenesis was induced by using the “StemPro Adipogenesis Differentiation Kit” (Cat. N. A1007001; Thermo Fisher Scientific, Waltham, MA, USA) following the manufacturer’s recommendations. The culture medium was changed twice weekly throughout the 14-day induction protocol. To assess the outcome of the differentiation protocol, cells were washed in PBS, fixed in 4% formaldehyde for 45 min, and subsequently stained with a filtered Oil Red O (O.R.O) solution (Cat. N. O0625; Sigma-Aldrich Co., St. Louis, MO, USA) 0.2% *w*/*v* in 60% 2-propanol (VWR International, Radnor, PA, USA) for 30 min at RT. O.R.O staining highlights the lipid vacuoles in the cytoplasm of adipocytes.

Osteogenic differentiation was investigated on hDMSCs seeded at a density of 5000 cells/cm^2^ in 24-well plates (Corning Incorporated, Corning, NY, USA). When cells reached 70% of confluence, they were cultured in the complete StemPro osteocyte differentiation basal medium (Cat. N. A1007201; Thermo Fisher Scientific, Waltham, MA, USA). The osteogenic medium was changed every 3–4 days throughout the 21-day differentiation protocol. To detect calcium deposits, cells were fixed in 4% formaldehyde for 15 min at RT, washed with deionized water, and stained with Alizarin Red S 1% *w*/*v* (Cat. N. A5533; Sigma-Aldrich Co., St. Louis, MO, USA).

Chondrogenic differentiation was induced on hDMSCs (5000 cells/cm^2^ in 24-well plates) when they reached 80% confluence. Cells were incubated with the complete StemPro chondrocyte differentiation basal medium (Cat. N. A1007101; Thermo Fisher Scientific, Waltham, MA, USA) for 28 days. During this period, the chondrogenic medium was changed every 3–4 days. Alcian Blue staining (Cat. N. A3157; Sigma-Aldrich Co., St. Louis, MO, USA) was used to detect the formation of the cartilage proteoglycans.

### 2.3. T-BHP and H_2_O_2_ Treatment

t-BHP (Cat. N. 8.14006; 70% solution in water) and H_2_O_2_ (Cat. N. H1009) were purchased from Sigma-Aldrich (Sigma-Aldrich Co., St. Louis, MO, USA). For all experiments, the cells were treated 24 h after seeding under standard culture conditions. In preliminary tests, the hDMSCs were exposed for 2 h to t-BHP diluted at different concentrations (10, 30, 50, and 100 μM) in L-DMEM or for 2 h to H_2_O_2_ at the concentrations 200, 300, and 400 μM. Then, experiments were performed with selected concentrations, 10, 30, and 50 μM for t-BHP and 200 μM for H_2_O_2_. Untreated cells were used as the control (CTR). At the end of the treatment, the medium was removed and replaced with a fresh and compound-free medium. After 48 h, cells were collected for all subsequent analyses.

### 2.4. Morphological Analysis

hDMSCs (3500 cells/cm^2^ in 24-well plates, Corning Incorporated, Corning, NY, USA-technical duplicate) were treated for 2 h with t-BHP (10, 30, 50, and 100 μM) or H_2_O_2_ (200, 300, and 400 μM). After 48 h from the end of the treatment, representative images of cells were acquired using the Leica Labovert FS Inverted Microscope (Wetzlar, Germany) with the Leica MC170 HD Imaging System Camera (Wetzlar, Germany).

### 2.5. Annexin V Detection Assay

hDMSCs (4500 cells/cm^2^ in 75 cm^2^ flasks, Corning Incorporated, Corning, NY, USA) were exposed for 2 h to t-BHP (30 or 50 μM) or to H_2_O_2_ 200 μM. After 48 h from the end of treatment, hDMSC viability, apoptosis, and necrosis were evaluated by using the commercial eBioscience^TM^ Annexin V-FITC apoptosis detention kit (Cat. N. BMS500FI; Invitrogen, Thermo-Fisher Scientific, Waltham, MA, USA). Following the manufacturer’s instructions, the cell suspension (0.25–1.0 × 10^7^ cells/mL) was washed in PBS and incubated in binding buffer 1X. A cell suspension (100 μL) was incubated with Annexin V-FITC (5 μL) for 10 min at RT in darkness and, subsequently, with propidium iodide (PI; 10 μL). Fluorescence was acquired by using the CytoFLEX S flow cytometer (Beckman-Coulter Inc., Brea, CA, USA) and data analysis was performed with FlowJo v10.8 software (Tree Star, Ashland, OR, USA). The four quadrants (Qs) in the scatterplots refer to living cells (Q4, Annexin V−/PI−), cells in the early stage of apoptosis (Q3, Annexin V+/PI−), necrotic cells (Q1, Annexin V−/PI+), and cells in the late stage of apoptosis (Q2, Annexin V+/PI+).

### 2.6. Bromodeoxyuridine (BrdU) Assay

To assess the cell proliferating rate after t-BHP/H_2_O_2_ treatments, the BrdU assay kit (Cat. N. 11647229001; Roche, Basel, Switzerland) was used. hDMSCs (3000 cells/cm^2^ in 96-well plates—Corning Incorporated, Corning, NY, USA—technical triplicate), were exposed for 2 h to t-BHP (10, 30, and 50 μM) or to H_2_O_2_ 200 μM. A negative control (medium without cells) was included. After 48 h from the end of the treatments, cells were incubated with BrdU labeling solution for 3 h at 37 °C. Subsequently, cells were fixed with FixDenat reagent for 30 min at RT. Then, hDMSCs were incubated with anti-BrdU antibody conjugated with peroxidase for 1 h and 30 min, followed by the incubation with the peroxidase substrate (5–10 min). The reaction was stopped by adding H_2_SO_4_ 1 M (Cat. N. 339741; Sigma-Aldrich Co., St. Louis, MO, USA) to each well. The absorbance at 450 nm was recorded with the Wallac 1420 Victor2 Multilabel Counter (Perkin Elmer, Waltham, MA, USA). hDMSC proliferation was expressed as the percentage of BrdU incorporated in treated cells compared with untreated (CTR) cells (set to 100%) ± standard deviation (SD).

### 2.7. RNA Extraction, RT-PCR, and Real-Time PCR

To evaluate the gene expression, hDMSCs (7000 cells/cm^2^ in 25 cm^2^ flasks, Corning Incorporated, Corning, NY, USA) were treated for 2 h with t-BHP (10, 30, and 50 μM) or H_2_O_2_ 200 μM. After 48 h from the end of the treatments, RNA was isolated by using the RNeasy mini kit (Cat. N. 74104; QIAGEN, Hilden, Germany) and digested with RNase-free Deoxyribonuclease I (Cat. N. 79254; RNase-free DNase set, QIAGEN, Hilden, Germany).

Afterwards, RNA was retrotranscribed into cDNA using the iScript^TM^ RT Supermix (Cat. N. 1708840; Bio-Rad Laboratories, Inc., Hercules, CA, USA). To confirm the success of the retrotranscription reaction, amplification and amplicon detection of the *glyceraldehyde 3-phosphate dehydrogenase* (*GAPDH*) gene were performed, as previously described [33,34].

For each experimental condition, 25 ng of cDNA was amplified in technical triplicates by using the SsoAdvanced Universal SYBR Green Supermix (Cat. N. 1725271; Bio-Rad Laboratories, Hercules, CA, USA) and the Bio-Rad CFX96 real-time PCR detection system (Bio-Rad Laboratories, Hercules, CA, USA), as previously described [33,35]. Gene expression was quantified by using CFX Manager Software version 3.1 (Bio-Rad Laboratories, Hercules, CA, USA) based on the “delta-delta CT method” [36]. The expression of genes involved in cell proliferation (*MKI67*), senescence (*CDKN1A*, *CDKN2A*, and *IL1B*), autophagy (*Beclin 1*—*BECN1*, *autophagy related 7*—*ATG7* and *microtubule-associated protein 1 light chain 3 alpha*—*MAP1LC3A*), and antioxidant response (*superoxide dismutase 1*—*SOD1* and *glutathione-disulfide reductase*—*GSR*), was normalized by using the two reference genes *GAPDH* and *hypoxanthine phosphoribosyl transferase 1* (*HPRT1*).

*GAPDH*, *HPRT1*, *CDKN1A*, and *CDKN2A* primers were purchased from Bio-Rad (20×, Bio-Rad Laboratories, Hercules, CA, USA); the other sequences were designed using the Primer Blast tool (https://www.ncbi.nlm.nih.gov/tools/primer-blast/, accessed on 6 April 2022) and Amplify4 software version 1 and were purchased from Sigma-Aldrich (Sigma-Aldrich Co., St. Louis, MO, USA). The primer sequences are listed in Table 1. For each gene, the normalized expression value of untreated cells (CTR) was set to 1, and gene expression values of treated cells were reported to that value. Data are expressed as fold change ± standard error of the mean (SEM).

### 2.8. Immunofluorescence Analysis of Proliferation and Cytoskeletal Markers

hDMSCs (5000 cells/cm^2^ on glass coverslips—Waldemar Knittel Glasbearbeitungs GmbH, Braunschweig, Germany—technical duplicate) were treated for 2 h with t-BHP (10, 30, and 50 μM) or with H_2_O_2_ 200 μM. After 48 h from the end of treatment, cells were fixed with 4% formaldehyde for 15 min, and then washed with PBS-Tween 0.25% (Cat. N. P2287; Sigma-Aldrich Co., St. Louis, MO, USA). Cells were permeabilized with PBS-Triton X-100 0.25% (Cat. N. X100; Sigma-Aldrich Co., St. Louis, MO, USA) and sodium citrate 10 mM (Sigma-Aldrich Co., St. Louis, MO, USA) for 15 min at RT. A solution containing 4% bovine serum albumin (BSA) (Cat. N. A9647; Sigma-Aldrich Co., St. Louis, MO, USA) in PBS-0.3% Triton X-100 was used to reduce non-specific antibody binding. To detect the proliferation marker Ki-67, the senescent-associated marker Lamin B1, the DNA damage marker phospho-H2AX (γH2AX), and the intermediate filament protein, vimentin, cells were incubated for 3 h at RT with the primary antibodies: anti-Ki-67 (Cat. N. TA336566; OriGene Technologies, Rockville, MD, USA), anti-Lamin B1 (Cat. N. A16685; ABclonal, Woburn, MA, USA), anti- γH2AX (Cat. N. AP0687; ABclonal, Woburn, MA, USA), and anti-vimentin (Cat. N. CS 5741; Cell Signaling Technology, Danvers, MA, USA). Then, cells were incubated for 1 h at RT using the following secondary antibodies: anti-rabbit Alexa Fluor 488 (Cat. N. A32731), anti-rabbit Alexa Fluor 555 (Cat. N. A32794), and anti-mouse Alexa Fluor 555 (Cat. N. A32727; Thermo-Fisher Scientific, Waltham, MA, USA). Both primary and secondary antibodies were used at 1:200 dilution in 2% BSA in PBS- 0.15% Triton X-100. To detect F-actin, hDMSCs were incubated with phalloidin-FITC (Cat. N. P5282; Sigma-Aldrich Co., St. Louis, MO, USA). Nuclei were counterstained with NucBlue^®^ Fixed Cell ReadyProbes^®^ Reagent (DAPI, Cat. N. R37606; Molecular Probes^TM^, Life Technologies-Thermo-Fisher Scientific, Waltham, MA, USA). The prolong antifade diamond mountant (Cat. N. P36965; Life Technologies-Thermo-Fisher Scientific, Waltham, MA, USA) was used to mount the slides. Images were acquired by using the Nikon Inverted Microscope Eclipse Ti2-E (Nikon Instruments, Melville, NY, USA) equipped with a digital sight camera DS-Qi2 (Nikon Instruments, Melville, NY, USA) through NIS-Elements software version 5.

The fluorescence intensity was quantified by using Fiji ImageJ software 2.1.0/1.53c [37], as described by Vidoni et al., 2019 [38], or by using NIS-Elements Advance Research software version 5. For Ki-67, fluorescence was quantified in 20 microscopic fields for each experimental condition and the mean fluorescence value was normalized to the mean area of nuclei. Data are presented as the normalized integrated density (IntDen) ± SEM. For cytoskeletal filaments, the mean fluorescence values of actin and vimentin proteins were measured independently for each experimental sample, and their ratio was calculated. Fluorescence was quantified in 8 random microscopic fields for each experimental condition. Data are presented as the ratio of IntDen actin/IntDen vimentin ± SD. For γH2AX and Lamin B1 staining, fluorescence was quantified in 5 random fields for each experimental condition. For γH2AX, data are reported as the mean of the total fluorescence intensity (FI) normalized to the total nuclear area ± SEM; for Lamin B1, data are presented as the normalized IntDen ± SEM.

### 2.9. Nuclear Dimension and Shape Factor Analysis

Nuclear dimensions of untreated (CTR) and t-BHP or H_2_O_2_-treated hDMSCs were assessed as previously described [39]. Briefly, DAPI images were analyzed using ImageJ 1.53 software. Images were transformed into an 8-bit image before being blurred (Process ⟶ Smooth: ×20 times). To identify the nuclear area, the threshold was automatically adjusted (Process ⟶ Binary ⟶ Make Binary) and near nuclei were separated to analyze them individually (Process ⟶ Binary ⟶ Watershed). The nuclear area was quantified by analyzing particles between 0 and ∞ (Analyze ⟶ Analyze Particles [size: 0–∞]). For each condition, 5 random images were analyzed. Data obtained are shown as the mean of the area of nucleus (μm^2^) ± SEM. The nuclear shape factor was measured by using NIS-Elements Advance Research software version 5. Data are reported as the mean of the nuclear shape factor ± SEM.

### 2.10. SA-β-Gal Assay

SA-β-Gal activity was evaluated by using the SA-β-Gal commercial kit (Cat. N. 9860; Cell Signaling Technology, Danvers, MA, USA). Briefly, hDMSCs (3500 cells/cm^2^ in a 24-well plate—Corning Incorporated, Corning, NY, USA—technical duplicate) were treated for 2 h with t-BHP (10, 30, and 50 μM) or with H_2_O_2_ 200 μM. After 48 h from the end of the exposure, cells were fixed and stained overnight according to the manufacturer’s instructions. Positive (blue) and negative (not colored) cells were manually counted in each condition in 5 random fields (10 fields/condition) using the Leica Labovert FS inverted microscope (Leica Microsystems, Wetzlar, Germany). Data are expressed as the percentage of SA-β-Gal-positive cells ± SD.

### 2.11. Cellular ROS Assay

To evaluate the cellular ROS production, the 2′,7-dichlorofluorescin diacetate (DCFDA)—cellular ROS assay kit was used (Cat. N. ab113851; Abcam Limited, Cambridge, UK). The assay is based on the oxidation of the non-fluorescent compound DCFDA by intracellular ROS into the fluorescent molecule 2′,7′-dichlorofluorescein (DCF). The fluorescence of DCF is detected by using a plate reader with excitation and emission of 485 nm and 535 nm, respectively. Briefly, hDMSCs (10,000 cells/cm^2^ in 96-black well plates with clear flat bottom, technical triplicate) were cultured for 24 h in HyClone Dulbecco’s modified Eagle medium with high glucose and without phenol red (H-DMEM, Cat. N. SH30284.01; Thermo-Fisher Scientific, Waltham, MA, USA), supplemented with 10% FBS and antibiotics (1% penicillin/streptomycin solution). Subsequently, cells were incubated for 30 min in standard condition with the cell permeant reagent DCFDA 10 μM. After removing the excess of DCFDA, hDMSCs were treated for 2 h with t-BHP (10, 30, and 50 μM) or with H_2_O_2_ 200 μM. Untreated cells were used as a control (CTR) and each experimental condition was performed in technical triplicate. A negative control without DCFDA was included. The fluorescence at 535 nm was acquired with the Enspire 2300 multi-mode microplate reader (Perkin Elmer, Waltham, MA, USA) immediately after the end of the treatment. For each measure, the value of ROS production was calculated as follows: FI 535 nm of test agent—FI 535 nm of untreated cells. Results are expressed as the mean fluorescence intensity ± SD.

### 2.12. Statistical Analysis

Data were analyzed by using GraphPad Prism software version 10 (GraphPad Software, San Diego, CA, USA). One-way ANOVA and post hoc Tukey’s test or Kruskal–Wallis test with post hoc Dunn’s test were used. Data are reported as mean ± SD or mean ± SEM. A *p*-value < 0.05 was considered statistically significant.

## 3. Results

### 3.1. hDMSC Characterization

**Figure 1:**
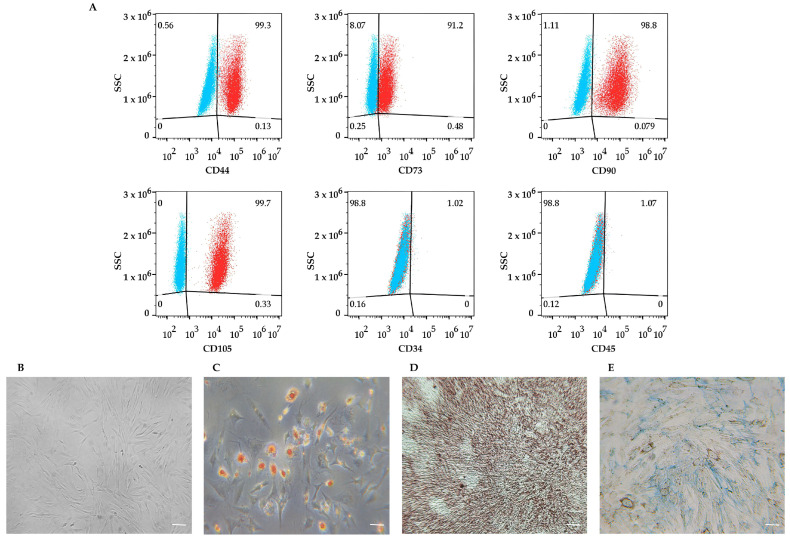
Characterization of human dermal mesenchymal stem cells (hDMSCs). (**A**) Flow cytometry-based immunophenotypic analysis. Cells expressed CD44, CD73, CD90, and CD105 mesenchymal surface markers and were negative for hematopoietic CD34 and CD45 ones. Blue and red clouds represent unstained cells (control) and cells stained with specific antibody, respectively. (**B**–**E**) Morphology and trilineage differentiation potential of hDMSCs: (**B**) hDMSCs undifferentiated; (**C**) adipocytes containing intracellular lipid vacuoles stained red by Oil Red O (O.R.O) solution; (**D**) osteocytes containing calcium deposits stained red by Alizarin Red S solution; (**E**) chondrocytes containing cartilage proteoglycans stained in blue with Alcian Blue solution. Scale bars: 50 μm.

hDMSCs were positive for MSC surface markers (CD44, CD73, CD90, and CD105) and were negative for the hematopoietic ones (CD34 and CD45) (Figure 1A).

Moreover, hDMSCs showed the MSC fibroblast-like shape (Figure 1B) and exhibited adipogenic, osteogenic, and chondrogenic differential ability (Figure 1C–E).

### 3.2. Viability of Treated hDMSCs

**Figure 2:**
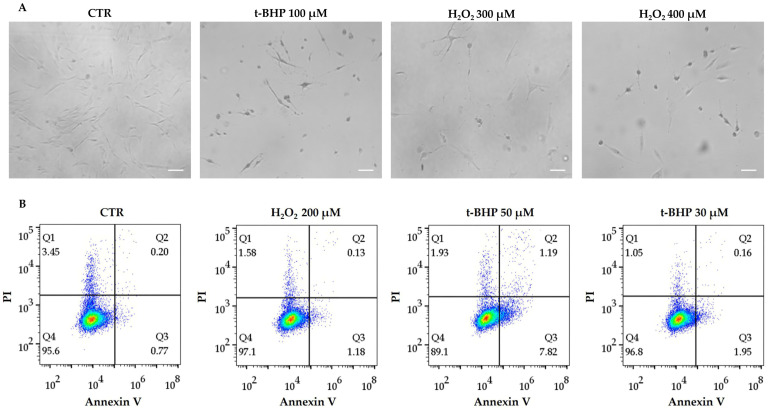
Viability of human dermal mesenchymal stem cells (hDMSCs) 48 h after the end of tert-butyl hydroperoxide (t-BHP) or hydrogen peroxide (H_2_O_2_) treatments. (**A**) Representative images of hDMSCs treated for 2 h with t-BHP 100 μM or H_2_O_2_ 300 and 400 μM, or untreated (CTR). The Leica Labovert FS inverted microscope integrated with a Leica MC170 HD imaging system camera was used to capture the images. Scale bars: 50 μm. (**B**) Viability of hDMSCs evaluated using the Annexin V/propidium iodide (PI) staining assay. hDMSCs were treated for 2 h with t-BHP (30 and 50 μM) or with H_2_O_2_ 200 μM. Untreated hDMSCs were used as control (CTR). Quadrants (Q) show the percentage of necrotic (Q1), late apoptotic (Q2), early apoptotic (Q3), and viable cells (Q4).

To evaluate the possible cytotoxic effects of t-BHP and H_2_O_2_, hDMSCs were preliminary treated with a wide range of concentrations and analyzed 48 h after the end of treatment using optical microscopy. The highest concentration of t-BHP (100 μM) as well as H_2_O_2_ at 300 and 400 μM concentrations were found to be cytotoxic; compared with the control cells (CTR), treated cells completely lost their shape and showed a tendency to detach from the plastic support (Figure 2A). Therefore, all these concentrations were excluded in subsequent experiments. 

Additionally, to investigate the effects of both t-BHP 30 and 50 μM, and H_2_O_2_ 200 μM on hDMSC viability, an Annexin V-PI staining assay was performed to identify the percentage of living apoptotic and necrotic cells. After 48 h from the end of treatment, no significant differences in the viability of hDMSCs treated with t-BHP 30 μM or H_2_O_2_ 200 μM were observed compared with untreated cells (CTR) (Figure 2B, lower left quadrant, Q4). Moreover, the hDMSCs were negative for Annexin V staining, had an apoptosis marker (upper and lower right quadrants, Q2 and Q3), and showed a percentage of necrotic cells (upper left quadrant, Q1) comparable to CTR (Figure 2B). Comparable results were observed in cells treated with t-BHP 50 μM, although 7.8% of early apoptotic cells were detected (Figure 2B). Based on all these results, the concentrations of t-BHP 10, 30, and 50 μM and H_2_O_2_ 200 μM were selected for all subsequent experiments.

### 3.3. Effects of t-BHP on Proliferative Ability of hDMSCs

**Figure 3:**
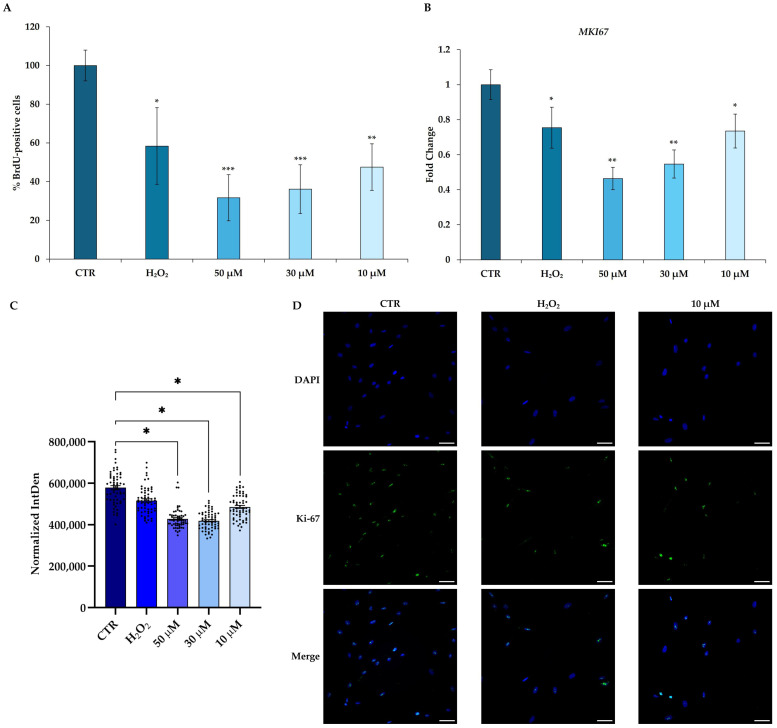
Proliferative ability of human dermal mesenchymal stem cells (hDMSCs) 48 h after the end of tert-butyl hydroperoxide (t-BHP) treatment. hDMSC proliferative ability was assessed in untreated (CTR) cells and in hDMSCs treated for 2 h with t-BHP (10, 30, and 50 μM) or hydrogen peroxide (H_2_O_2_) 200 μM. Untreated cells were used as control (CTR). (**A**) Proliferation analysis of hDMSCs by BrdU assay. Histograms represent the mean percentage of BrdU-positive cells ± standard deviation (SD). *n* = 3; one-way ANOVA: * *p* < 0.05, ** *p* < 0.01, and *** *p* < 0.001 vs. CTR. (**B**) Gene expression analysis of *proliferation marker protein Ki-67* (*MKI67*) in hDMSCs. Data were normalized using two housekeeping genes (*glyceraldehyde 3-phosphate dehydrogenase*—*GAPDH* and *hypoxanthine phosphoribosyl transferase 1*—*HPRT1*); the normalized expression value of CTR was set up to 1, and all other gene expression values were reported to that sample. Data are reported as normalized fold change ± standard error of the mean (SEM); *n* = 3. One-way ANOVA: * *p* < 0.05 and ** *p* < 0.01 vs. CTR. (**C**,**D**) Immunofluorescence evaluation of Ki-67 in hDMSCs. (**C**) Fluorescence intensity of Ki-67 protein was measured by Fiji software 2.1.0/1.53c, and it was reported as IntDen (integrated density). Data are presented as the mean IntDen calculated in 20 selected fields normalized to the mean area of nuclei (normalized IntDen ± SEM); *n* = 3; one-way ANOVA: * *p* < 0.05. (**D**) Representative images of hDMSCs treated with t-BHP 10 μM, H_2_O_2_ 200 μM, or untreated. Cells were immunostained with anti-Ki-67 antibody (green signal) and DAPI was used to counterstain nuclei (blue signal). Scale bars: 50 μm.

To assess the effect of t-BHP on hDMSC proliferation, cells were incubated with a BrdU labeling solution after 48 h from the end of treatment with t-BHP (10, 30, and 50 μM) or H_2_O_2_ 200 μM. Cell proliferation was assessed by measuring BrdU incorporation, a thymidine analogue, in the newly synthesized DNA strands during cell division. t-BHP treatment statistically reduced the percentage of BrdU-positive cells compared with CTR in a dose-dependent manner (Figure 3A). H_2_O_2_ also significantly reduced the percentage of BrdU-positive cells, but to a lesser extent than t-BHP (Figure 3A).

Moreover, to confirm the proliferation data, the expression of the *MKI67* gene was investigated. Both treatments significantly reduced the expression of *MKI67* mRNA, with a trend similar to that observed in the BrdU assay (Figure 3B). According to these results, the data obtained with the immunofluorescence assay showed a significant decrease in the number of Ki-67-positive cells (green signal) after treatment with t-BHP, already evident at the lowest tested concentration of 10 μM compared with CTR (Figure 3C,D and Figure S1). In addition, the weaker effect of H_2_O_2_ on the reduction in proliferation of hDMSCs compared with t-BHP was here confirmed (Figure 3C,D and Figure S1).

### 3.4. Effects of t-BHP on Morphology and Cytoskeleton Organization of hDMSCs

**Figure 4:**
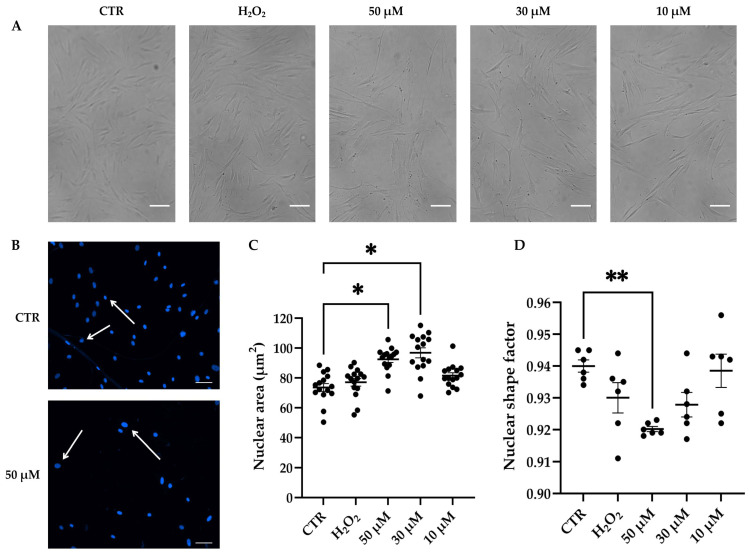
Morphology in human dermal mesenchymal stem cells (hDMSCs) 48 h after the end of tert-butyl hydroperoxide (t-BHP) treatment. hDMSCs were treated for 2 h with t-BHP (10, 30, and 50 μM) or hydrogen peroxide (H_2_O_2_) 200 μM. Untreated cells were used as control (CTR). (**A**) Morphological analysis of hDMSCs. Scale bars: 50 μm. (**B**,**C**) Analysis of nuclear size in hDMSCs. hDMSC nuclei were counterstained with NucBlue^®^ Fixed Cell ReadyProbes^®^ Reagent (DAPI) (blue signal). (**B**) Representative images of CTR cells and cells treated with 50 μM t-BHP acquired using a Nikon inverted microscope Eclipse Ti2-E and a digital sight camera DS-Qi2, through the imaging software NIS-Elements. White arrows highlight nuclei of different sizes. Scale bars: 25 μm. (**C**) The scatter plot represents the mean of the nuclei areas ± standard error of the mean (SEM) analyzed by using ImageJ 1.53 software. Each dot corresponds to an image containing at least 300 cells; *n* = 3. One-way ANOVA: * *p* < 0.05 vs. CTR. (**D**) The scatter plot represents the mean of nuclear shape factor ± SEM. Data were obtained by analyzing nuclei image with NIS-Elements Advance Research software; *n* = 3. One-way ANOVA: ** *p* < 0.01.

Cell morphology was evaluated in untreated and t-BHP- or H_2_O_2_-treated hDMSCs after 48 h from the end of the treatments. Both molecules were able to change hDMSC morphology that tended to appear enlarged and elongated (Figure 4A). Specifically, morphology alteration was more pronounced in cells treated with the highest t-BHP concentrations (30 and 50 μM) compared with those treated with the lower t-BHP concentration (10 μM) or with H_2_O_2_ 200 μM (Figure 4A).

Higher concentrations of t-BHP (30 and 50 μM) also induced a significant increase in the area of hDMSC nuclei compared with CTR, while treatment with H_2_O_2_ did not show evident effects on the nuclear dimension (Figure 4B,C). Moreover, the nuclear shape factor, a parameter used in imaging analysis to assess alterations in nuclear morphology, was investigated. Values deviating from 1 (nuclear circularity) indicate increasing nuclear irregularity. Data showed both t-BHP- or H_2_O_2_- treated cells exhibited a decreasing trend in this parameter. Such a reduction was statistically significant only in cells treated with 50 µM t-BHP compared with CTR, suggesting the presence of nuclear morphological alterations (Figure 4D).

**Figure 5:**
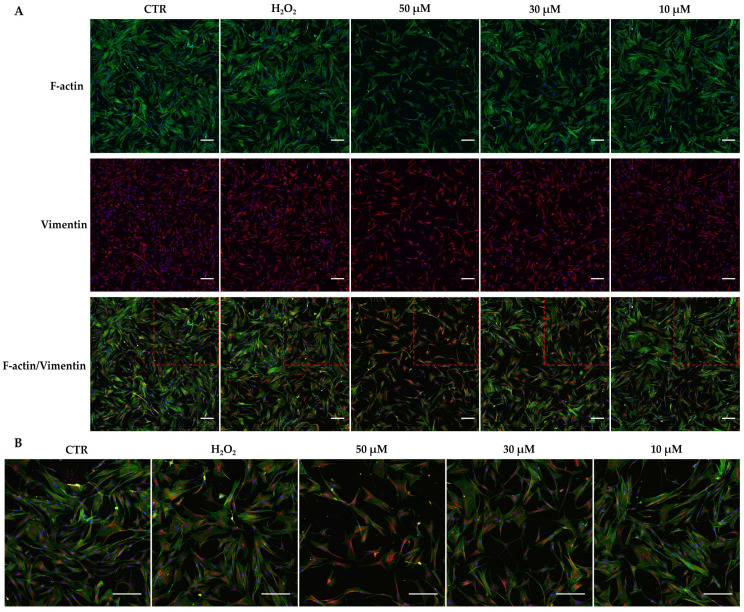
Immunofluorescence evaluation of cytoskeleton markers in human dermal mesenchymal stem cells (hDMSCs) 48 h after the end of tert-butyl hydroperoxide (t-BHP) treatment. (**A**) hDMSCs were treated for 2 h with t-BHP (10, 30, and 50 μM) or hydrogen peroxide (H_2_O_2_) 200 μM. Untreated cells were used as control (CTR). hDMSCs were stained with Phalloidin (green signal, specific for F-actin) and anti-vimentin antibody (red signal). DAPI was used to counterstain nuclei (blue signal). The ratio between actin filaments and vimentin is highlighted by the orange signal; an increase in the intensity of this color indicates a decrease in the ratio. The dotted red square indicates the area of the image that is magnified in (Panel **B**). Scale bars: 50 μm. (**B**) Magnification (dotted red square) of the images in (Panel **A**).

In addition, to evaluate the effects of t-BHP on cytoskeletal organization, both treated and untreated hDMSCs were stained with phalloidin (specific for F-actin) and with anti-vimentin antibody 48 h after the end of the treatment, and then analyzed by immunofluorescence. t-BHP induced a dose-dependent change in cytoskeleton organization, evidenced by a decrease in the F-actin/vimentin ratio (orange signal) (Figure 5). This decrease was statistically significant at the concentration of 50 μM t-BHP (Table 2). In contrast, H_2_O_2_ did not lead to a significant reorganization of the cytoskeleton (Figure 5 and Table 2).

### 3.5. t-BHP Induced a Senescence Phenotype of hDMSCs

**Figure 6:**
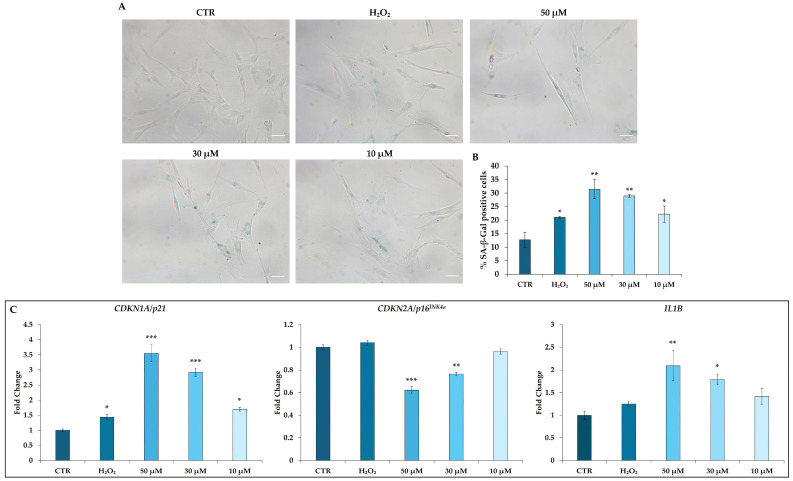
Evaluation of cellular senescence in human dermal mesenchymal stem cells (hDMSCs) 48 h after the end of tert-butyl hydroperoxide (t-BHP) treatment. hDMSCs were treated for 2 h with t-BHP (10, 30, and 50 μM) or hydrogen peroxide (H_2_O_2_) 200 μM, or untreated (CTR). (**A**,**B**) Senescence-associated β-galactosidase (SA-β-Gal) staining assay in hDMSCs. (**A**) Representative images of SA-β-Gal assay: positive cells are blue and negative cells are unstained. Scale bars: 50 μm. (**B**) Histograms represent the percentage of SA-β-Gal positive cells manually counted ± standard deviation (SD). *n* = 3; one-way ANOVA: * *p* < 0.05 and ** *p* < 0.01 vs. CTR. (**C**) Gene expression analysis of senescent markers *cyclin-dependent kinase inhibitor 1A* (*CDKN1A* or *p21*), *cyclin-dependent kinase inhibitor 2A* (*CDKN2A* or *p16^INK4a^*), and *interleukin 1 beta* (*IL1B*) in hDMSCs. Data were normalized using two housekeeping genes (*glyceraldehyde 3-phosphate dehydrogenase*—*GAPDH* and *hypoxanthine phosphoribosyl transferase 1*—*HPRT1*); the normalized expression value of CTR was set up to 1, and all other gene expression values were reported to that sample. Data are reported as normalized fold change ± standard error of the mean (SEM); *n* = 3. One-way ANOVA: * *p* < 0.05, ** *p* < 0.01, and *** *p* < 0.001 vs. CTR.

hDMSCs were analyzed by the SA-β-Gal assay to evaluate the ability of t-BHP to modulate cell senescence (Figure 6A). After 48 h from the end of the treatment with t-BHP (10, 30, and 50 μM) or H_2_O_2_ 200 μM, the percentage of SA-β-Gal-positive cells was statistically higher in treated hDMSCs compared with CTR cells, with the highest increase observed at t-BHP concentrations of 30 and 50 μM (Figure 6B).

To further support the pro-senescence effect of t-BHP, the analysis of other senescence markers was evaluated.

In particular, the expressions of *CDKN1A* (alias *p21*) and *CDKN2A* (alias *p16^INK4a^*) genes were also measured. Both t-BHP and H_2_O_2_ induced a significant increase in *CDKN1A* expression compared with the CTR condition. In particular, the expression of this gene increased in a dose-dependent manner in cells treated with t-BHP; such an increase was higher at t-BHP concentrations of 30 and 50 μM compared with H_2_O_2_ treatment (Figure 6C). On the contrary, t-BHP induced a dose-dependent decrease in *CDKN2A* expression, while H_2_O_2_ seemed not to affect its expression (Figure 6C).

Among SASP factors, *IL1B* was selected to analyze its expression. Data reported in Figure 6C show the upregulation of the mRNA levels of *IL1B* in cells treated with 30 µM or 50 µM t-BHP compared with CTR. No difference in *IL1B* expression was observed in cells treated with H_2_O_2_ or 10 µM t-BHP (Figure 6C).

**Figure 7:**
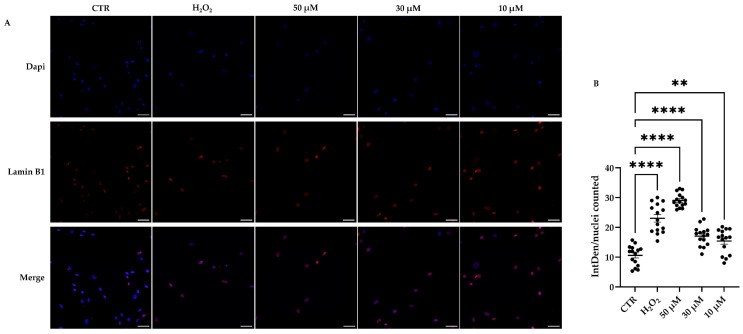
Immunofluorescence analysis of Lamin B1 in human dermal mesenchymal stem cells (hDMSCs) 48 h after the end of tert-butyl hydroperoxide (t-BHP) or hydrogen peroxide (H_2_O_2_) treatments. hDMSCs were treated for 2 h with t-BHP (10, 30, and 50 μM) or H_2_O_2_ 200 μM. Untreated cells were used as control (CTR). (**A**) Representative images of hDMSCs stained with anti-Lamin B1 antibody (red signal) and DAPI, used to counterstain nuclei (blue signal). Scale bars: 50 μm. (**B**) Quantification of Lamin B1 fluorescence by using NIS-Elements Advance Research software. The scatter plot represents the mean of fluorescence intensity (IntDen) normalized on the number of cells ± standard error of the mean (SEM). Each dot represents the number of fields analyzed in each condition. In each field, at least 300 nuclei were counted; *n* = 3. One-way ANOVA: ** *p* < 0.01 and **** *p* < 0.0001.

Finally, another senescence marker was performed. As shown in Figure 7A,B, both stressor agents increased the expression of Lamin B1 in treated hDMSCs compared with CTR. In particular, a dose-dependent enhancement in Lamin B1 expression was observed in cells treated with t-BHP (Figure 7B).

### 3.6. t-BHP Induced DNA Damage Response (DDR) of hDMSCs

**Figure 8:**
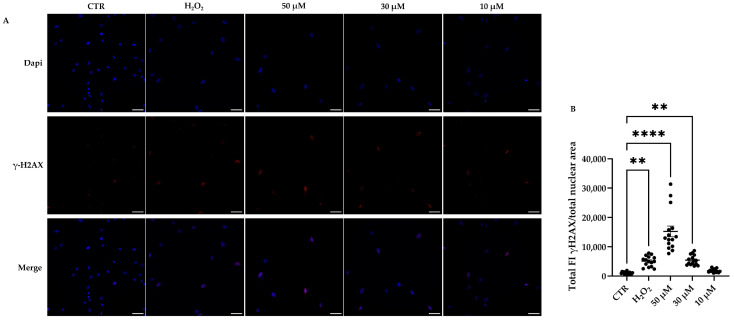
Immunofluorescence analysis of phosphorylated H2AX (γH2AX) in human dermal mesenchymal stem cells (hDMSCs) 48 h after the end of tert-butyl hydroperoxide (t-BHP) or hydrogen peroxide (H_2_O_2_) treatments. hDMSCs were treated for 2 h with t-BHP (10, 30, and 50 μM) or H_2_O_2_ 200 μM. Untreated cells were used as control (CTR). (**A**) Representative images of hDMSCs stained with anti-γH2AX antibody (red signal) and DAPI, used to counterstain nuclei (blue signal). Scale bars: 50 μm. (**B**) Quantification of γH2AX fluorescence by using NIS-Elements Advance Research software. The scatter plot represents the mean of total fluorescence intensity (FI) normalized on the total nuclear area ± standard error of the mean (SEM). Each dot represents the number of fields analyzed in each condition. In each field, at least 300 nuclei were counted; *n* = 3. One-way ANOVA: ** *p* < 0.01 and **** *p* < 0.0001.

Since senescence was shown to be induced by a DNA damage response (DDR), we evaluated the activation of the DDR pathway in hDMSCs treated with both H_2_O_2_- and t-BHP by analizing the γH2AX, a variant of the histone H2A. As shown in Figure 8, the expression of γH2AX was significantly increased in cells treated with H_2_O_2_ and with high concentrations of t-BHP, 30 and 50 µM.

### 3.7. Effects of t-BHP on Autophagy and Oxidative Stress in hDMSCs

**Figure 9:**
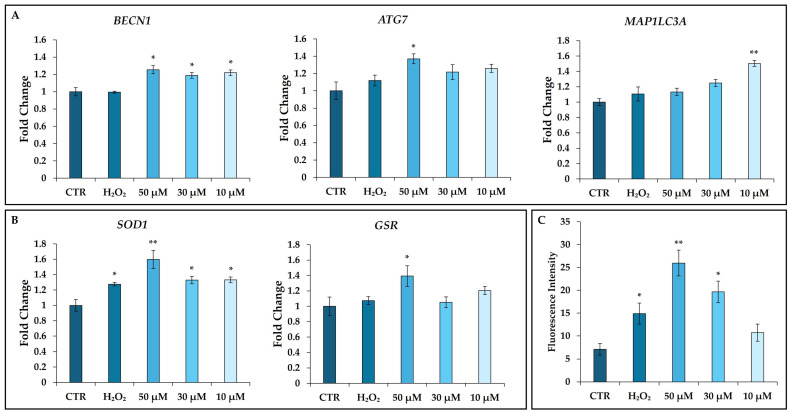
Evaluation of autophagy and oxidative stress pathways in human dermal mesenchymal stem cells (hDMSCs) 48 h after the end of tert-butyl hydroperoxide (t-BHP) treatment. hDMSCs were treated for 2 h with t-BHP (10, 30, and 50 μM) or hydrogen peroxide (H_2_O_2_) 200 μM, or untreated (CTR). (**A**,**B**) Gene expression analysis of autophagy-related markers and antioxidant genes in hDMSCs. The expression of genes involved in the (**A**) autophagy pathway *beclin 1* (*BECN1*), *autophagy related 7* (*ATG7*), and *microtubule-associated protein 1 light chain 3 alpha* (*MAP1LC3A*) or in the (**B**) oxidative stress pathway *superoxide dismutase 1* (*SOD1*) and *glutathione-disulfide reductase* (*GSR*) was evaluated. Data were normalized using two housekeeping genes (*glyceraldehyde 3-phosphate dehydrogenase*—*GAPDH* and *hypoxanthine phosphoribosyl transferase 1*—*HPRT1*); the normalized expression value of CTR was set up to 1, and all other gene expression values were reported to that sample. Data are reported as normalized fold change ± standard error of the mean (SEM); *n* = 3. One-way ANOVA: * *p* < 0.05 and ** *p* < 0.01 vs. CTR. (**C**) Reactive oxygen species (ROS) production in hDMSCs. The extent of ROS generation was evaluated in hDMSCs treated for 2 h with t-BHP (10, 30, and 50 μM) or H_2_O_2_ 200 μM, or without treatment (CTR) immediately after the end of the treatments. Histograms represent the mean fluorescence intensity at 535 nm ± standard deviation (SD). *n* = 3; One-way ANOVA: * *p* < 0.05 and ** *p* < 0.01 vs. CTR.

The involvement of two senescence-related pathways (autophagy and oxidative stress) was evaluated by the gene expression analysis of selected markers in hDMSCs untreated (CTR) or treated for 2 h with t-BHP (10, 30, and 50 μM) or H_2_O_2_ (200 μM). After 48 h from the end of the treatment, the mRNA levels of autophagy-related genes *BECN1*, *ATG7*, and *MAP1LC3A* were analyzed. The treatment with t-BHP significantly increased the expression of *BECN1* at all investigated concentrations compared with CTR (Figure 9A); while only the highest concentration of t-BHP (50 μM) significantly stimulated the expression of *ATG7* compared with CTR (Figure 9A). On the contrary, only the lowest concentration of t-BHP (10 μM) was able to significantly increase the expression of *MAP1LC3A* in hDMSCs compared with CTR (Figure 9A). H_2_O_2_ had no effect on the expression of the autophagy-related markers studied (Figure 9A).

Moreover, at the same experimental conditions, the effect of t-BHP and H_2_O_2_ on the expression of two antioxidant genes *SOD1* and *GSR* was analyzed. *SOD1* gene expression was significantly increased after both treatments. On the other end, only the concentration of 50 μM t-BHP statistically increased the expression of *GSR* (Figure 9B).

To further support the effect of t-BHP in inducing oxidative stress, cellular ROS levels were assessed by incubating hDMSCs with DCFDA for 30 min followed by a 2 h treatment with t-BHP (10, 30, and 50 μM) or H_2_O_2_ (200 μM), or no treatment (CTR). Inside the cell, DCFDA was deacetylated by cellular esterases to a non-fluorescent compound, which was oxidized by cellular ROS into DCF. Both t-BHP and H_2_O_2_ treatment induced a significant increase in ROS, more pronounced in cells treated with t-BHP 50 μM (Figure 9C).

## 4. Discussion

For years, MSCs have been used in regenerative medicine to treat various pathologies due to their differentiative and immunomodulatory properties. However, a treatment protocol that is always effective has not yet been identified, since the use of these cells is affected by several issues. For example, the in vitro expansion of MSCs, necessary to obtain an adequate number of cells to be transplanted, increases the probability to compromise their proliferative and differentiative potential and instead leads to cellular senescence [11,12,13].

Understanding the molecular pathways that are involved in senescence is crucial to block or reverse this process; this is one of the main purposes of regenerative medicine. To achieve this goal, it is essential to use a solid in vitro model of cellular senescence. Two methods are used to obtain senescent SCs in vitro: the expansion of the cells for several culture passages (a process that requires a long time) or the use of physical or chemical agents capable of inducing SIPS in cultured cells [15,17].

t-BHP is a molecule widely used to induce senescence in various cell types, including SCs [24,25,26,27,28,29,30,31].

In this research, we investigated the effects of different concentrations of t-BHP on hDMSCs, a promising model in cell-based therapy [5,6,7,8,9,10], to establish an in vitro model of SIPS. We also used H_2_O_2_, another well-known senescence inducer and already studied in this cell model, for comparing purposes.

To this end, the concentrations of both t-BHP and H_2_O_2_ as well as the exposure times were initially chosen after a careful study of the literature. Several t-BHP-based protocols for senescence induction have employed varying concentrations, exposure times, and the presence or absence of a post-treatment recovery period [24,28,29,30,40,41,42]. These differences in cell treatment protocols may depend on the sensitivities of the different cellular models to t-BHP treatment. Instead, the concentrations of H_2_O_2_ to be employed were chosen based on the literature data related to DMSCs [22,23] and on our previous study, where the effects of H_2_O_2_ were investigated on MSCs isolated from other human tissues [19]. Therefore, based on our published method and following the experimental approach reported by Li and colleagues [42], we treated cells for 2 h, followed by a 48 h recovery period in fresh and compound-free medium. Preliminary tests were performed using t-BHP or H_2_O_2_ at the final concentrations of 10, 30, 50, and 100 μM and 200, 300, and 400 μM, respectively, to verify the potential cytotoxic action of these molecules. The toxicity of t-BHP 100 μM and of H_2_O_2_ 300 and 400 μM was demonstrated by morphological analysis, which highlighted that both molecules at these concentrations induced cell damage. After confirming the non-toxicity of selected concentrations of t-BHP (10, 30, and 50 μM) and H_2_O_2_ (200 μM) by using Annexin V-PI staining assay, further analyses were carried out to study several markers that characterize the senescent phenotype.

Firstly, the proliferative ability of treated and untreated hDMSCs was assessed; both molecules led to a significant reduction in the BrdU-positive cell percentage. Interestingly, the reduction in proliferation was more evident in hDMSCs treated with t-BHP than H_2_O_2_, as further indicated by the significant decrease in expression of *MKI67* and its Ki-67 related protein, a positive regulator of the cell cycle.

A reduction in the number of hDMSCs was also evident in the morphological analysis, where t-BHP treatment, especially at the highest concentration (50 μM), also caused a loss of the cell typical fibroblast-like phenotype, which appeared more enlarged [13,19]. Moreover, hDMSCs appeared “spread” on the substrate and this could be associated with a pro-senescent structural alteration of the nucleus that tends to increase its size and exhibit shape abnormalities [43,44,45]. These changes were particularly evident at the highest concentrations of t-BHP.

Alterations in cell morphology, nuclear dimension, and shape are reasonably due also to an altered organization of the cyto- and nucleoskeleton. It is known that senescent cells showed an altered expression and organization of cytoskeletal filaments resulting in an F-actin/vimentin ratio reduction [43,44]. Our findings corroborate this observation; in fact, the pronounced increase in the orange signal associated especially with the concentration of t-BHP 50 μM was indicative of a decrease in the F-actin/vimentin ratio. This result could be explained by the progressive loss of actin filaments (green signal) in favor of an increase in the number and thickness of the vimentin filaments (red signal), which could prevent the collapse of the cellular structure and give the cell greater resistance to mechanical stress [43,44]. Notably, H_2_O_2_ treatment also led to an alteration in cell morphology and cytoskeleton organization in hDMSCs, but less pronounced than t-BHP at higher concentrations tested.

Interestingly, we also observed an unexpected increase in Lamin B1 expression following t-BHP- or H_2_O_2_-induced treatment. Lamin B1, an essential protein of nuclear lamina, belongs to the intermediate filament family. It provides structural support to the nuclear envelope maintaining nuclear shape and plays a crucial role in nuclear function and organization [46]. A reduction in Lamin B1 expression has been observed in senescent cells and is commonly considered a marker of senescence [46,47,48]. This loss is associated with impairments in nuclear membrane integrity and alterations in gene expression due to changes in chromatin organization [46,49]. However, consistent with our data, other studies have reported conflicting results regarding the regulation of Lamin B1 expression in senescent cells. For instance, it was also observed that the treatment of fibroblasts with H_2_O_2_ or L-buthionine sulfoximine (BSO), which depletes the glutathione pool, led to the accumulation of Lamin B1 via p38 mitogen-activated protein kinase (MAPK) activation [50]. The high levels of Lamin B1, in turn, influenced nuclear morphology and triggered senescence [50]. In line with these observations, Dreesen and colleagues showed that Lamin B1 overexpression impairs fibroblast proliferation and induces senescence, effects that are further exacerbated by a simultaneous reduction in LMNA/C expression [51]. Collectively, these findings suggest that maintaining appropriate levels of Lamin B1 expression is essential for preserving cellular homeostasis, while both its upregulation and downregulation may contribute to the induction of senescence [46]. Notably, it was observed that the overexpression of Lamin B1 impairs the proper recruitment of the tumor suppressor p53-binding protein 1 (53BP1) to DNA damage sites, leading to persistent DNA damage and increased sensitivity to double-strand breaks (DSBs) [52]. The accumulation of irreparable DBSs is strictly associated with cellular senescence and induces a DDR mediated by the ataxia–telangiectasia mutated/ataxia–telangiectasia and Rad3-related (ATM/ATR) kinases [53]. Both kinases promote the phosphorylation of the H2AX (γH2AX), a variant of the histone H2A, recruiting and organizing a network of DDR proteins such as 53BP1 to repair DNA damage [54]. In our model, the concomitant increase in γH2AX and Lamin B1 suggests that t-BHP treatment induces DNA damage that remains unrepaired due to Lamin B1 accumulation, which interferes with the DDR by preventing the recruitment of 53BP1 to damaged sites. Similar effects were observed in H_2_O_2_-treated cells, although they seem less pronounced compared with those induced by higher concentrations of t-BHP, suggesting that t-BHP is stronger than H_2_O_2_ in inducing cellular senescence in this cell model.

The persistent DNA damage induces cell cycle arrest and, consequently, a block in cell proliferation, a typical hallmark of all senescent cells [13], as clearly observed in t-BHP-treated hDMSCs.

This process could be triggered by the activation of two molecular pathways, p53/p21 and p16^INK4a^/pRB, involving the two cyclin-dependent kinase inhibitor proteins (CDKIs) p21 and p16^INK4a^, respectively. In particular, the activation of the first pathway is strictly dependent on p53 protein, which is activated by DNA damage, but also in response to other cellular stress conditions such as the high production of ROS. p53 activation leads to the expression of p21, which acts by blocking the activation of the CDK4/CDK6-cyclin D1, thus preventing the transition from the G1 phase to the S phase and cell cycle progression [12,55]. This occurred in hDMSCs exposed to t-BHP or H_2_O_2_, where the expression of *p21* gene was significantly higher than that of CTR cells and showed a t-BHP dose-dependent trend. This data, together with the decrease in Ki-67 expression, could explain the reduction in cell proliferation observed in treated cells.

Unlike *p21*, *p16^INK4a^* expression showed a significant dose-dependent decrease in cells treated with t-BHP. This result contrasts with the well-established finding that p16^INK4a^ expression is markedly upregulated in senescent cells, as well as during physiological aging and in age-related diseases [56]. However, the expression of p16^INK4a^ is complex and tightly regulated by several mechanisms [57,58]. It is known that *p16^INK4a^* transcription is modulated through a negative feedback loop involving pRB; in fact, the phosphorylation of pRB promotes the translocation of E2F, which induces the expression of the S phase genes, as well as *p16^INK4a^*. Subsequently, p16^INK4a^ protein inhibits CDK4/6 and enhances the hypo-phosphorylation of pRB, ultimately leading to the downregulation of the *p16^INK4a^* transcript [58]. This molecular mechanism could explain the *p16^INK4a^* mRNA decrease in our model, following the initial upregulation of the corresponding protein. However, we cannot be certain of this regulatory mechanism of *p16^INK4a^* as we have not evaluated its protein expression. Therefore, we cannot rule out the involvement of additional regulatory pathways that may suppress *p16^INK4a^* mRNA expression and contribute to this unexpected result.

In addition to senescence-associated changes in morphology, cytoskeleton organization, and proliferation, in our model, hDMSCs treated with the highest concentration of t-BHP also expressed the pro-inflammatory *IL1B*, suggesting the acquiring of SASP, which contributed to reinforce senescence in an autocrine/paracrine manner [59,60]. Moreover, hDMSCs treated with t-BHP or H_2_O_2_ also showed an increased activity of lysosomal enzyme SA-β-Gal, a hydrolase that cleaves the terminal residues of β-D-galactose present in numerous substrates, such as lactose molecules, keratan sulfates, and sphingolipids. Under normal conditions, this enzyme is active at a pH of 4.0, while, in senescent cells, a SA-β-Gal that works at a pH of 6.0 accumulates and therefore allows to discriminate senescent cells from young cells [61]. The process that leads to the accumulation of this enzyme is still unclear, but it is probably linked to the formation of larger and more active lysosomal vesicles in senescent cells, an event due to an alteration of autophagic process [62,63]. There is great controversy about the role of autophagy in the senescence process. Some authors have described an inhibition of the autophagic process with consequent accumulation of damaged proteins and organelles in senescent cells. Therefore, the restoration of autophagic activity could promote the survival of the senescent cells, allowing them to proliferate [64]. On the contrary, other researchers have attributed autophagy a senescence-promoting role. Gerland and colleagues, for example, have described a high formation of autophagic inclusions in senescent fibroblasts, in which the activity of SA-β-Gal is concentrated [63]. Instead, Chang and collaborators observed a correlation between the senescent process induced in bone marrow-derived MSCs (BMSCs) following exposure to high glucose levels and autophagy, describing a greater expression of the genes involved in this pathway, thus attributing it a primary role in the onset of senescence [64]. Similarly, in this study, the treatment with t-BHP led to an increase in the expression of *BECN1*, *ATG7*, and *MAP1LC3A*, all genes involved in the formation of autophagic vesicles, supporting a role of autophagy in the senescence process of hDMSCs [65]. Moreover, in different studies, autophagy seemed to be involved in the alteration of nuclear size, associated with a progressive alteration of Lamin B1, an intermediate filament characteristic of the nucleoskeleton [43,44,48]. As above discussed, this action on the nucleus was observed in hDMSCs treated with t-BHP, confirming its role in hDMSC senescence. In contrast, the expression of autophagic genes did not change in cells exposed to H_2_O_2_, indicating a minor effect of this molecule to induce senescence or a different mechanism of action that does not involve the autophagic pathway. However, the role of the autophagic process in senescence of hDMSCs remains to be clarified.

Cellular senescence can be related also to oxidative stress conditions. As is well known, oxidative stress arises from an imbalance between ROS production and antioxidant defense, which leads to ROS-dependent damages to numerous components of the cell, from entire organelles such as mitochondria to single macromolecules like DNA [66]. DNA damage leads to the activation of the p53 pathway, which, to safeguard the state of the cell, blocks its proliferation through the activation of p21 [13,43]. Moreover, ROS can act on actin monomers by reducing their ability to polymerize through the addition of glutathione on Cys374 residues [44]. Both t-BHP and H_2_O_2_ are key molecules involved in the induction of a condition of oxidative stress, as confirmed in this study by the increase in ROS production. However, the greater expression of the *SOD1* gene in hDMSCs treated with both molecules highlighted the attempts of cells to counteract oxidative stress. SOD1 is an enzyme with antioxidant activity that catalyzes the conversion of the superoxide anion (O_2_^•−)^ in O_2_ and H_2_O_2_ and whose expression increases in the presence of oxidative stress [67]. The expression of *SOD1* was higher in hDMSCs exposed to the concentration of 50 μM of t-BHP, confirming its stronger effect compared with H_2_O_2_. At this concentration, a significant increase in the expression of *GSR* gene, another antioxidant enzyme, was also observed in t-BHP-treated cells compared with that of CTR cells, while no effect in the expression of this enzyme was observed in H_2_O_2_-exposed cells. GSR is an enzyme that catalyzes the reduction of glutathione disulfide (GSSG) to glutathione (GSH), the substrate of the enzyme glutathione peroxide (GPX) that uses it to convert a molecule of H_2_O_2_ into water [68], suggesting that the t-BHP-mediated GSH depletion induced the modulation of GSH-cycle enzymes. Collectively, our results suggest that t-BHP is a stronger pro-senescence molecule compared with H_2_O_2_ for inducing SIPS in hDMSCs. Molecular mechanisms underlying t-BHP- or H_2_O_2_-induced senescence in hDMSCs were not investigated in our research, but other studies have analyzed the mechanism of action of t-BHP or H_2_O_2_ in MSCs independently. For instance, in human umbilical cord-derived MSCs, H_2_O_2_ has been shown to induce a DNA damage response through a complex interplay between the mTORC1/p70S6K pathway and the heterochromatin organization, thus leading to the SIPS phenotype [69]. On the other hand, a recent study has proposed that t-BHP induces cell senescence in NPMSCs by modulating a competitive endogenous RNA network that involved the circSPG21, miR-217, and NAD-dependent protein deacetylase sirtuin-1 (SIRT1) axis [41]. Further research is needed to compare the molecular mechanism involved in t-BHP- or H_2_O_2_-mediated SIPS in MSCs.

## 5. Conclusions

Our results suggest that the highest tested concentration of t-BHP induced SIPS in hDMSCs. This compound triggered oxidative stress in these cells, as evidenced by the increase in ROS production. Although the enhanced expression of *SOD* and *GSR* preserved cells from death, they still experienced cellular alterations and DNA damage. Such impairment, in turn, could enhance the expression of p21 via p53, inducing a block in cell proliferation, changes in cellular and nuclear morphology, and cytoskeletal reorganization, thus leading to a senescent phenotype.

However, it is necessary to extend our observations to longer time points (7–14 days) following hDMSC treatment. This will allow to determine whether the cells have entered a senescence program that progresses towards a chronic senescence state, or whether the observed cycle arrest represents only a transient response to stress-induced DNA damage. In the latter case, the cells would be able to resume proliferation once the t-BHP–induced damage is repaired. For this purpose, it would be interesting to further investigate the expression of Lamin B1 and p16^INK4a^ at a longer recovery period after the t-BHP-treatment, as their behavior in our MSC model appears to contrast with their widely recognized roles in cellular senescence. For instance, on the one hand, the increase in the expression of Lamin B1 observed in this study can compromise the DNA repair mechanisms [52,53], favoring the appearance of a senescent phenotype; on the other hand, its increase could be linked to an increase in proliferative capacity and a delay in the onset of senescence [70,71]. Similarly, a decrease in p16^INK4a^ expression could be associated with increased cell proliferation and attenuation of the SASP, and is a common event in human cancers, where it has been related to senescence bypass [72].

Moreover, the analysis of IL-6 and IL-8 expression, SASP markers commonly used to define stable/chronic senescence [73,74], could provide new insights into the regulatory mechanisms of t-BHP in the senescence process, offering further evidence of its ability to induce a SIPS model.

Finally, it is essential to explore the t-BHP role across different MSC types, to determine whether the effects we observed are broadly shared or restricted to specific MSC subtypes.

In conclusion, although our findings, together with previously published results, suggest that t-BHP may represent a promising compound for inducing SIPS in hDMSCs, further investigations are necessary. This SIPS model could be used in several fields of regenerative medicine as a valuable tool to study the senescence process and evaluate the efficacy of senolytic compounds.

## Figures and Tables

**Table 1:** Primer details and sequences.

Gene	Entrez Gene ID *	Left Primer	Right Primer	Bio-Rad Unique Assay ID	A.L. (bp) ^$^
*Glyceraldehyde 3-phosphate dehydrogenase* (*GAPDH*)	2597	-	-	qHsaCED0038674	117
*Hypoxanthine phosphoribosyl transferase 1* (*HPRT1*)	3251	-	-	qHsaCID0016375	90
*Proliferation marker protein Ki-67* (*MKI67*)	4288	tcagactccatgtgcctgag	ttgtcctcagccttctttgg	-	134
*Cyclin-dependent kinase inhibitor 1A* (*CDKN1A or p21*)	1026	-	-	qHsaCID0014498	159
*Cyclin-dependent kinase inhibitor 2A* (*CDKN2A or p16^INK4a^*)	1029	-	-	qHsaCED0056722	86
*Interleukin 1 beta* (*IL1B*)	3553	agccatggcagaagtacctg	cctggaaggagcacttcatct	-	116
*Beclin 1* (*BECN1*)	8678	aaccagatgcgttatgccca	tccattccacgggaacactg	-	148
*Autophagy related 7* (*ATG7*)	10533	agcagctcatcgaaagccat	ttggcaaaaagcgatgagcc	-	241
*Microtubule-associated protein 1 light chain 3 alpha* (*MAP1LC3A*)	84557	ttggtcaagatcatccggcg	cctgggaggcgtagaccata	-	163
*Superoxide dismutase 1* (*SOD1*)	6647	ggtgtggccgatgtgtctat	cacctttgcccaagtcatct	-	112
*Glutathione-disulfide reductase* (*GSR*)	2936	cccgatgtatcacgcagtta	aaaccctgcagcatttcatc	-	129

* ID—identification number; ^$^ A.L. (bp)—amplicon length (base pair).

**Table 2:** Ratio of mean fluorescence intensities of actin and vimentin.

Sample	IntDen Actin/IntDen Vimentin ± SD
CTR	1.61 ± 0.09
H_2_O_2_ 200 μM	1.52 ± 0.08
t-BHP 50 μM	1.11 ± 0.04 *
t-BHP 30 μM	1.49 ± 0.05
t-BHP 10 μM	1.59 ± 0.04

CTR—control; H_2_O_2_—hydrogen peroxide; t-BHP—tert-butyl hydroperoxide; integrated density—IntDen; standard deviation—SD; one-way ANOVA: *, *p* < 0.05.

## Data Availability

The data used to support the findings of this study are available from the corresponding author upon request.

## Supplementary Materials

The following supporting information can be downloaded at: https://www.mdpi.com/article/10.3390/cells14191563/s1, Figure S1. Representative images at lower magnification of immunofluorescence staining for Ki-67 in hDMSCs 48 h after the end of tert-butyl hydroperoxide (t-BHP) or hydrogen peroxide (H_2_O_2_) treatments. hDMSCs were treated for 2 h with t-BHP 10 μM or H_2_O_2_ 200 μM. Untreated cells were used as control (CTR). Cells were stained with anti-Ki-67 antibody (green signal) and DAPI was used to counterstain nuclei (blue signal). Scale bar: 50 µm.

## Abbreviations

The following abbreviations are used in this manuscript:

SC	Stem cells
hMSCs	Human mesenchymal stem cells
DMSCs	Dermal mesenchymal stem cells
M1	Type 1 macrophages
M2	Type 2 macrophages
GVHD	Graft-versus-host disease
SASP	Senescence associated secretory phenotype
H_2_O_2_	Hydrogen peroxide
t-BHP	Tert-butyl hydroperoxide
h	Hours
GSH	Glutathione
ROS	Reactive oxygen species
CDKN1A	Cyclin-dependent kinase inhibitor 1A
P53	Tumor protein P53
SA-β-Gal	Senescence-associated β-galactosidase
MMP1	Matrix metalloproteinase 1
MMP3	Matrix metalloproteinase 3
SERPINB2	Serpin family B member 2
IL-1A	Interleukin 1A
EGF	Epidermal growth factor
IL-8	Interleukin 8
NSCs	Neuronal stem cells
MKI67	Marker protein Ki-67
CDKN2A	Cyclin-dependent kinase inhibitor 2A
CCND1	Cyclin D1
pRb	Retinoblastoma protein
NPMSCs	Nucleus pulposus-derived MSCs
IL1B	Interleukin 1B
IL-6	Interleukin 6
JNK	Jun N-terminal kinase
L-DMEM	Dulbecco’s modified Eagle’s medium—1 g/L of glucose
FBS	Fetal bovine serum
CO_2_	Carbon dioxide
PBS	Phosphate buffered saline
RT	Room temperature
FITC	Fluorescein isothiocyanate
PE	Phycoerythrin
PerCP	Peridinin chlorophyll
O.R.O	Oil Red O
CTR	Control
PI	Propidium iodide
Q	Quadrant
BrdU	Bromodeoxyuridine
SD	Standard deviation
GAPDH	Glyceraldehyde 3-phosphate dehydrogenase
BECN1	Beclin 1
ATG7	Autophagy related 7
MAP1LC3A	Microtubule-associated protein 1 light chain 3 alpha
SOD1	Superoxide dismutase 1
GSR	Glutathione-disulfide reductase
HPRT1	Hypoxanthine phosphoribosyl transferase 1
SEM	Standard error of the mean
BSA	Bovine serum albumin
γH2AX	Phospho-H2AX
IntDen	Integrated density
FI	Fluorescence intensity
H-DMEM	Dulbecco’s modified Eagle medium with high glucose and without phenol red
DCFDA	Dichlorofluorescin diacetate
DCF	Dichlorofluorescein
DDR	DNA damage response
BSO	L-buthionine sulfoximine
p38 MAPK	P38 mitogen-activated protein kinase
53BP1	tumor suppressor p53-binding protein 1
DSBs	double-strand breaks
ATM/ATR	Ataxia–telangiectasia mutated/ataxia–telangiectasia and Rad3-related
CDKIs	Cyclin-dependent kinase inhibitor proteins
CDK4	Cyclin dependent kinase 4
CDK6	Cyclin dependent kinase 6
BMSCs	Bone marrow-derived mesenchymal stem cells
O2^•−^	Superoxide anion
GSSG	Glutathione disulfide
GPX	Glutathione peroxide
SIRT1	sirtuin-1
SIPS	Stress-induced premature senescence
