# Supplementary figures and images for: Tert-Butyl Hydroperoxide in Human Adult Mesenchymal Stem Cells Isolated from Dermis: A Stress-Induced Premature Senescence Model
Source: Cells. 2025 Oct 8;14(19):1563. doi: 10.3390/cells14191563 (PMC12523526; doi:10.3390/cells14191563)

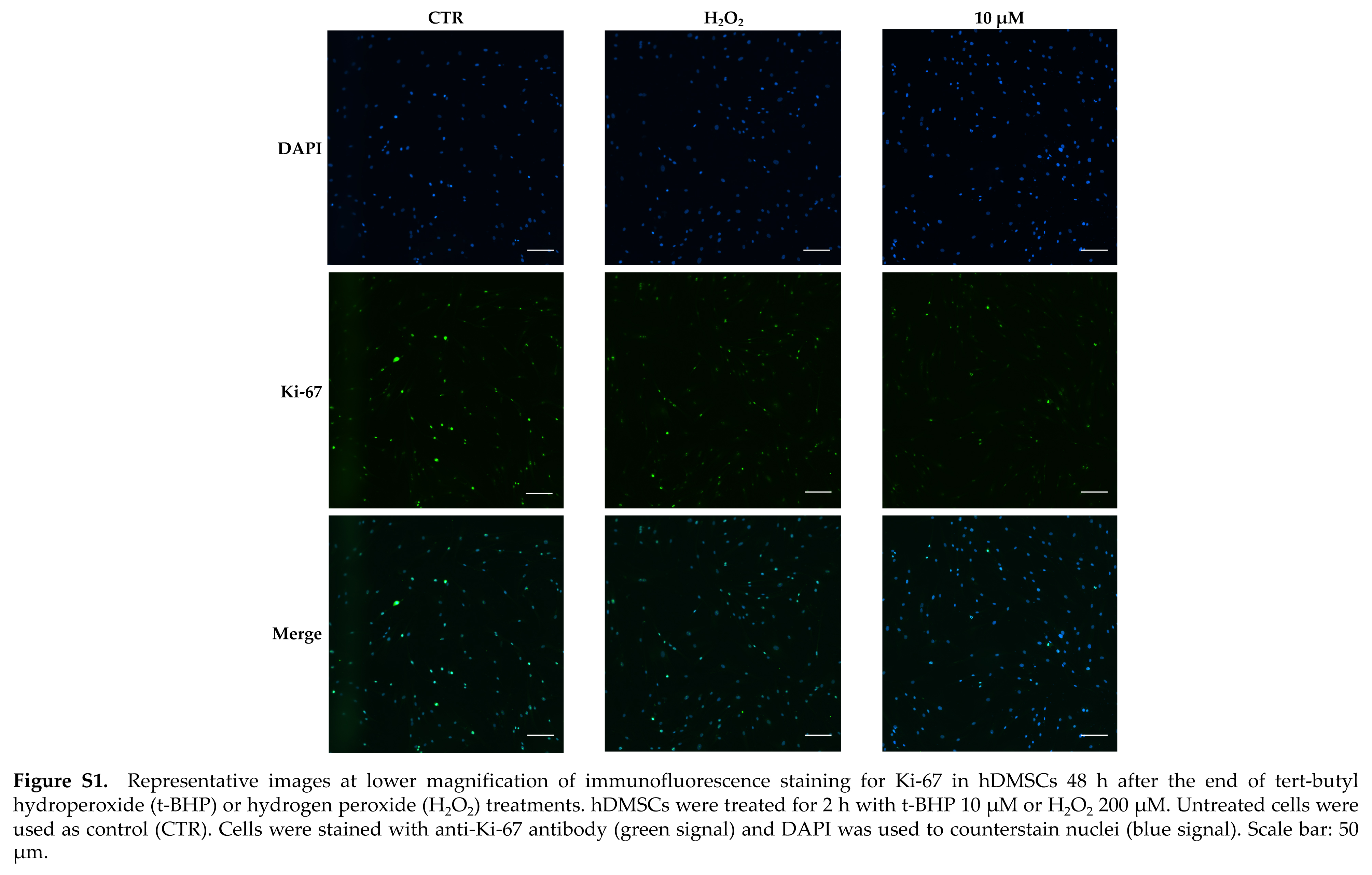

Supplement: Supplementary file 1 [file cells-14-01563-s001.zip › Figure S1 v2.jpg]
